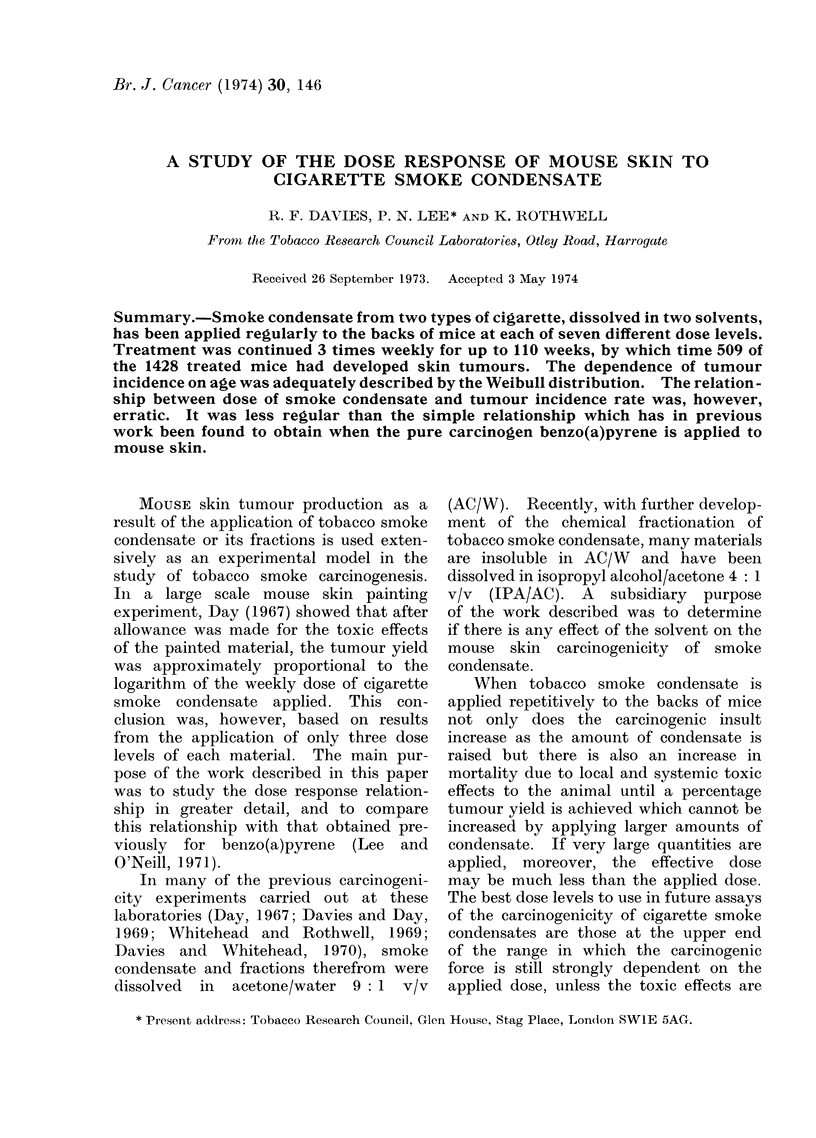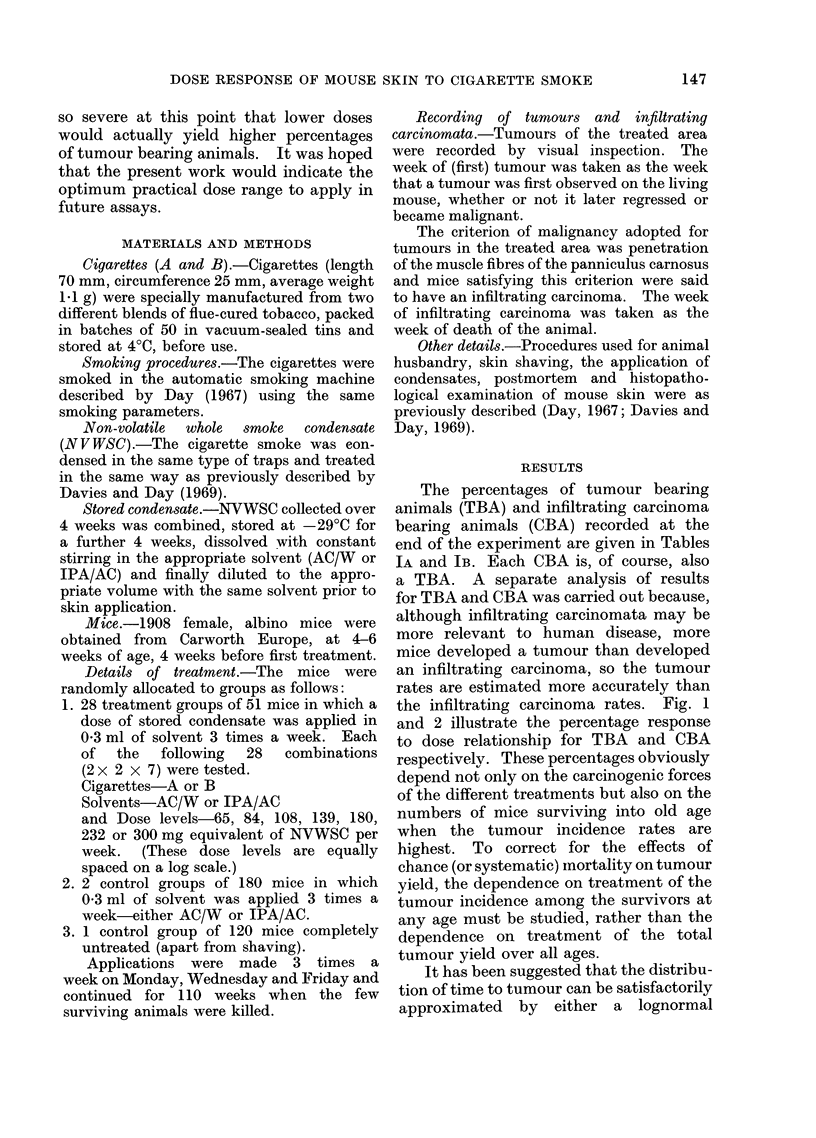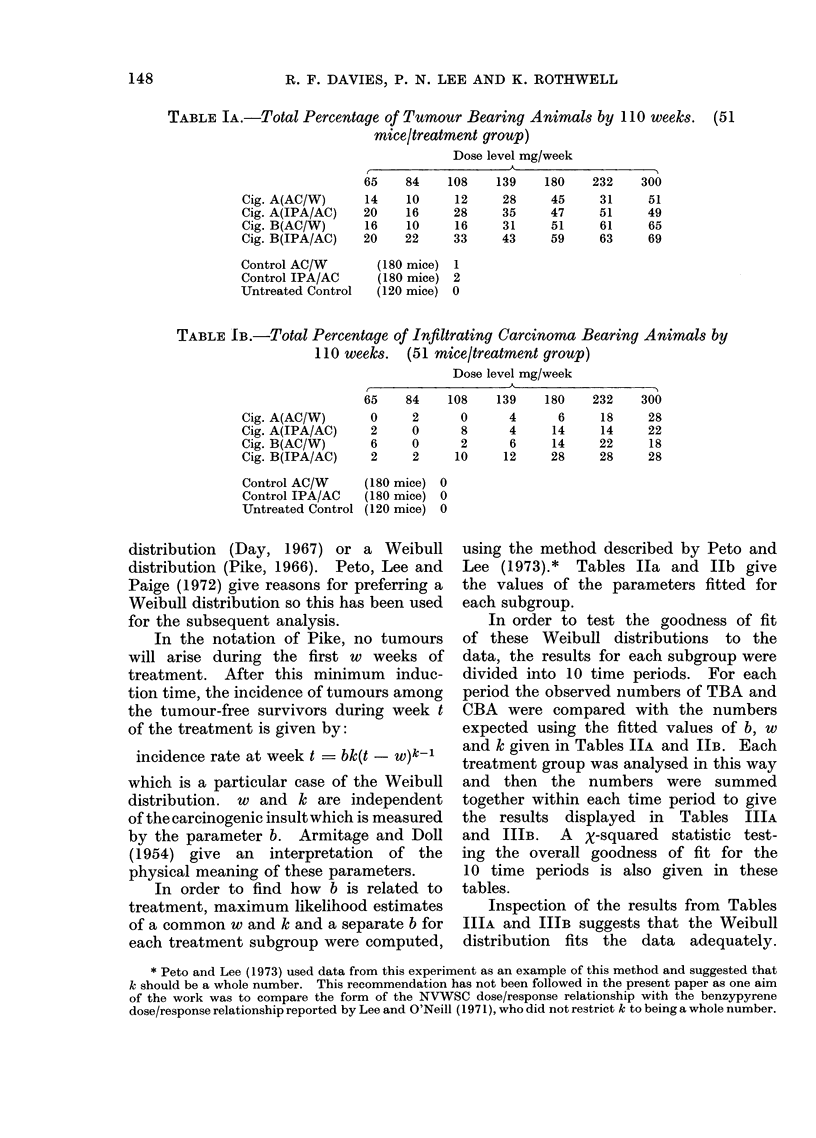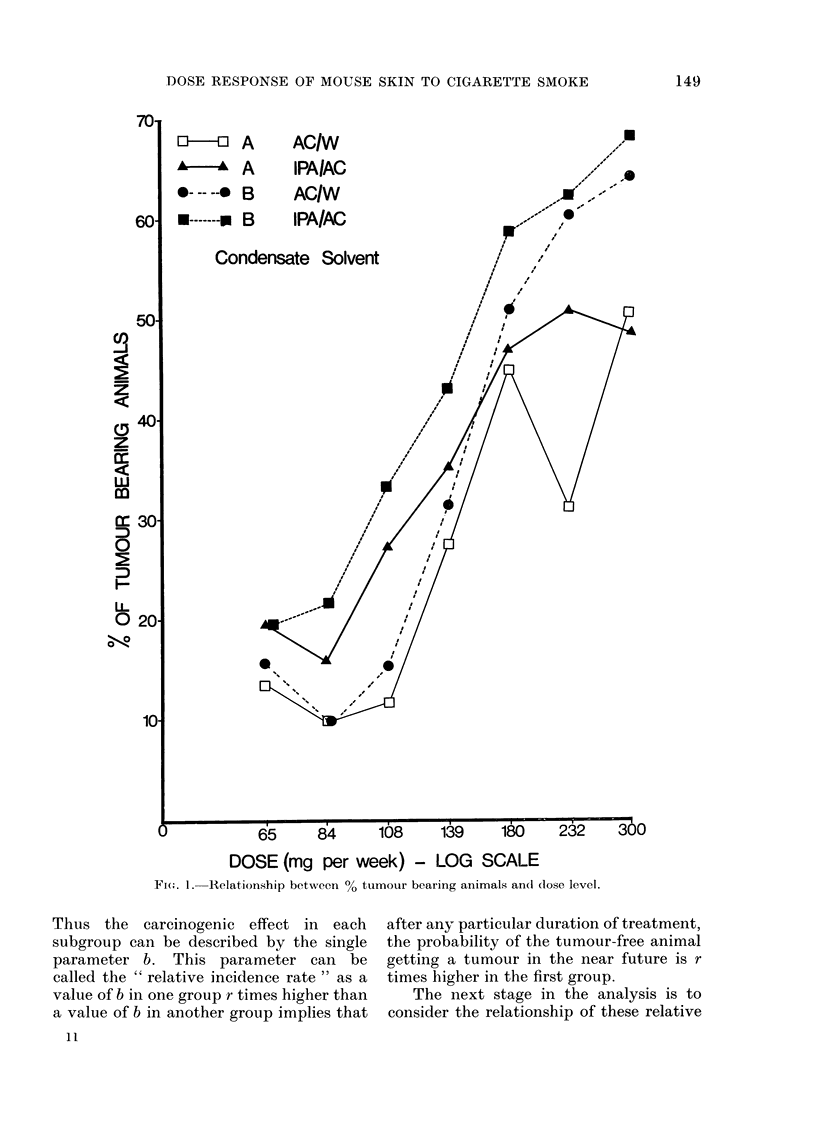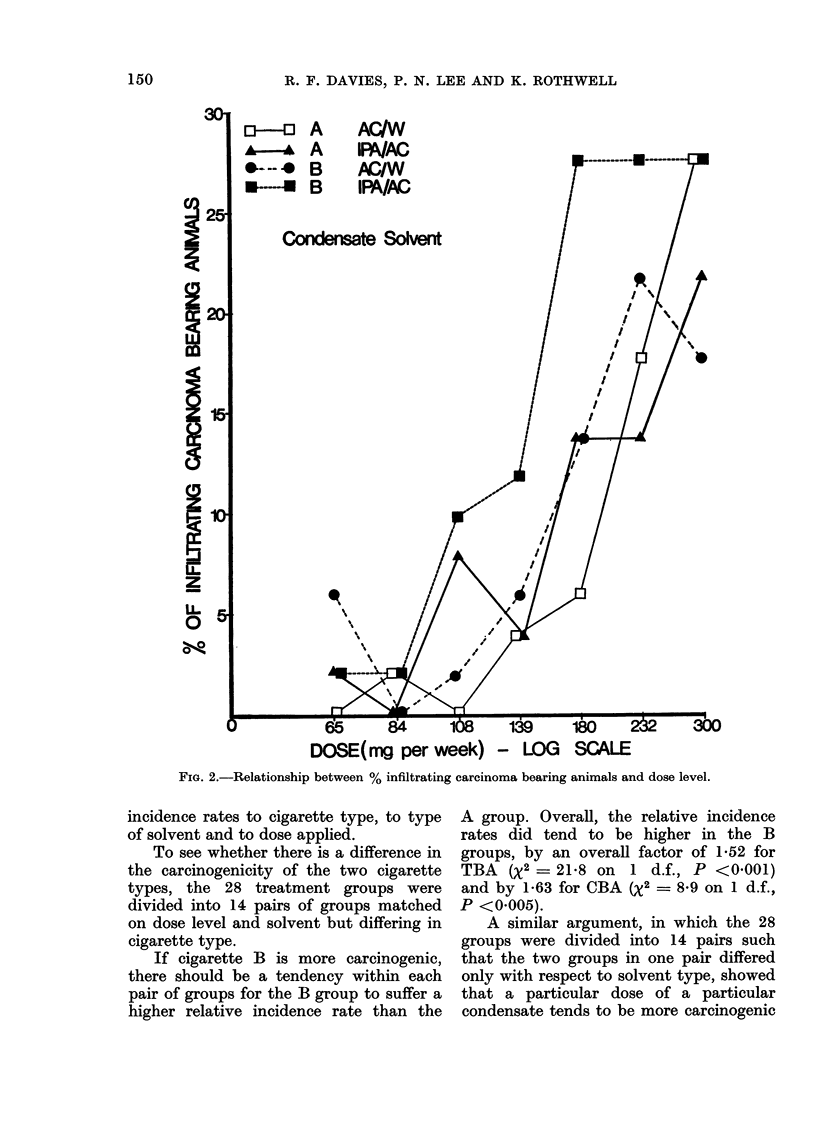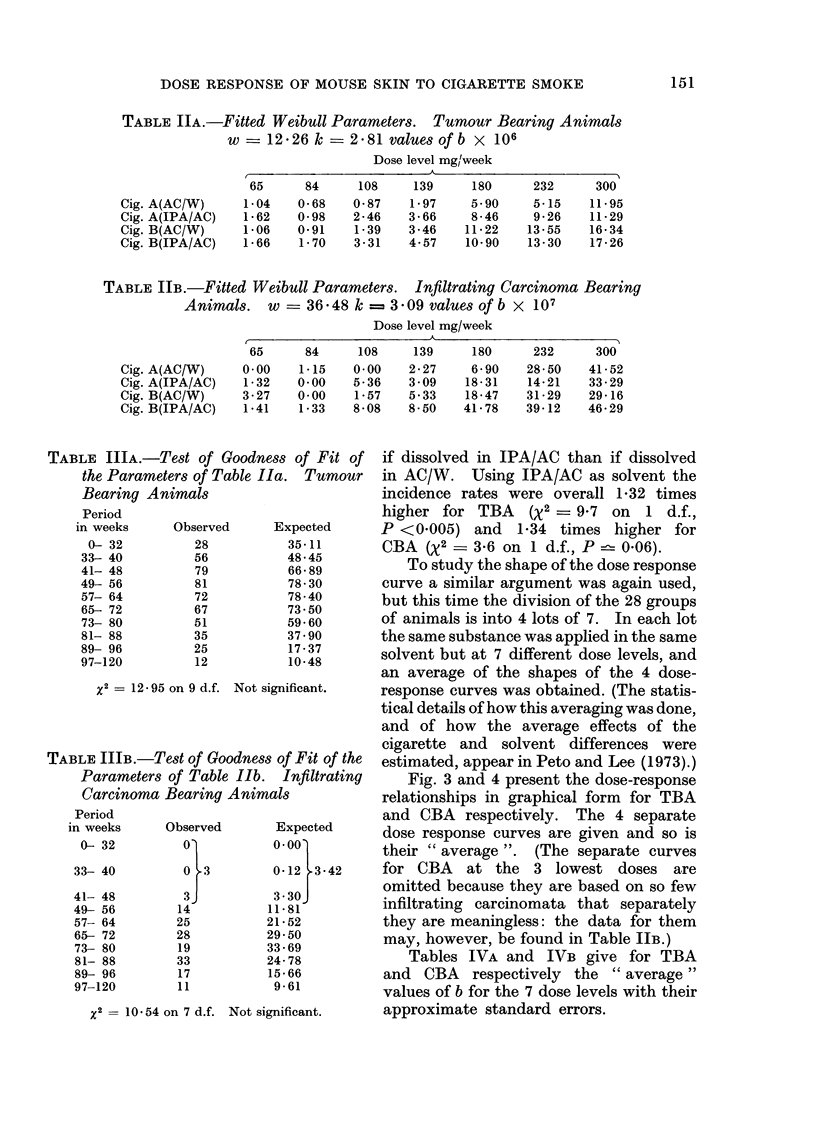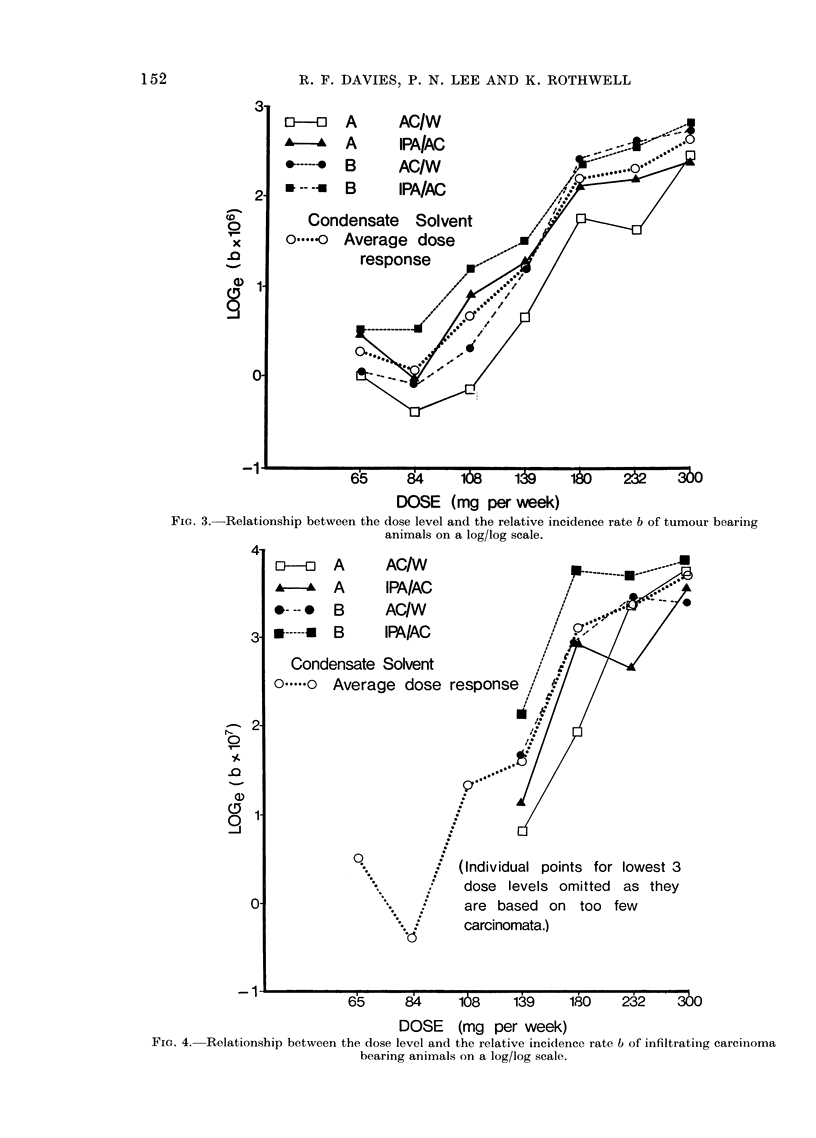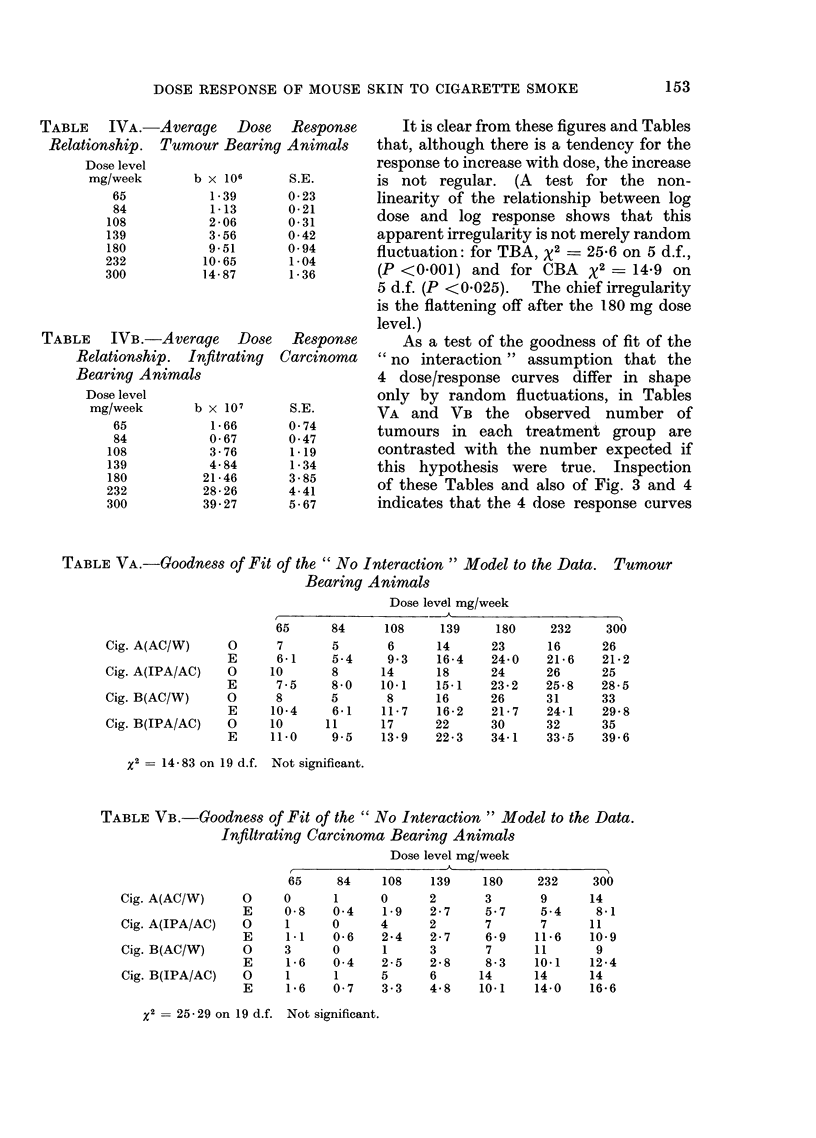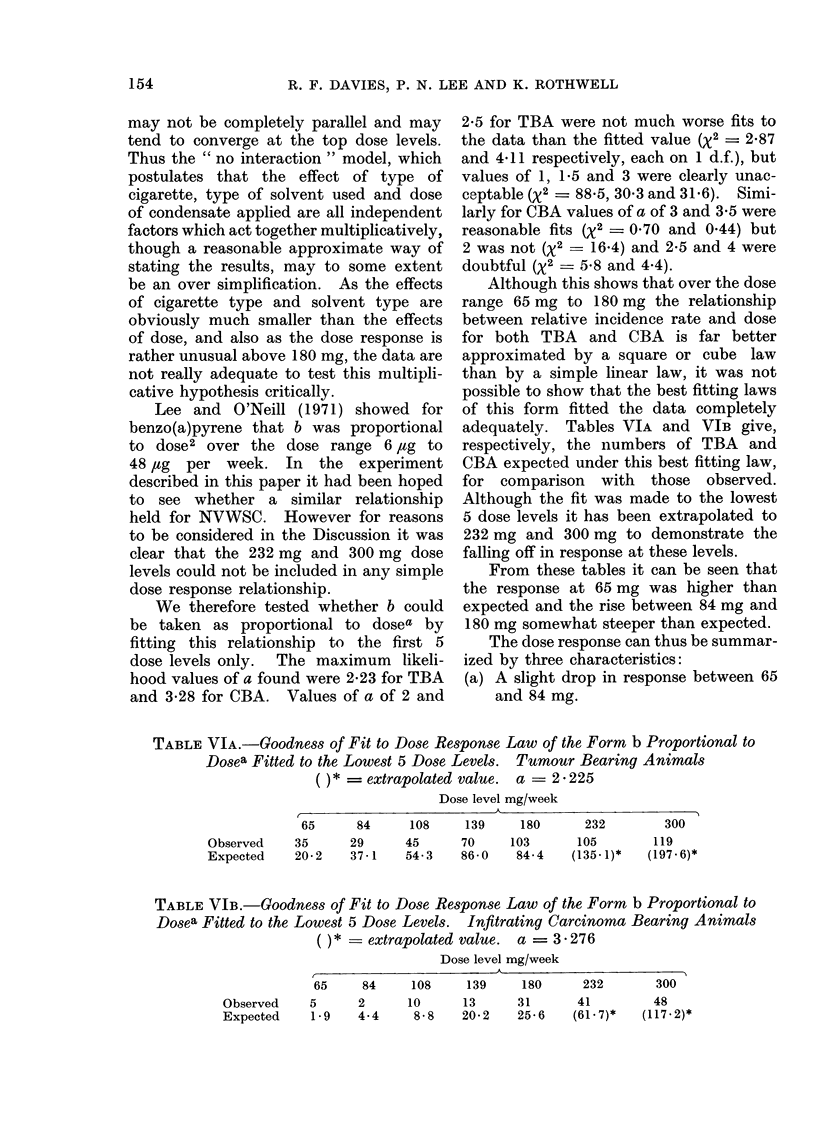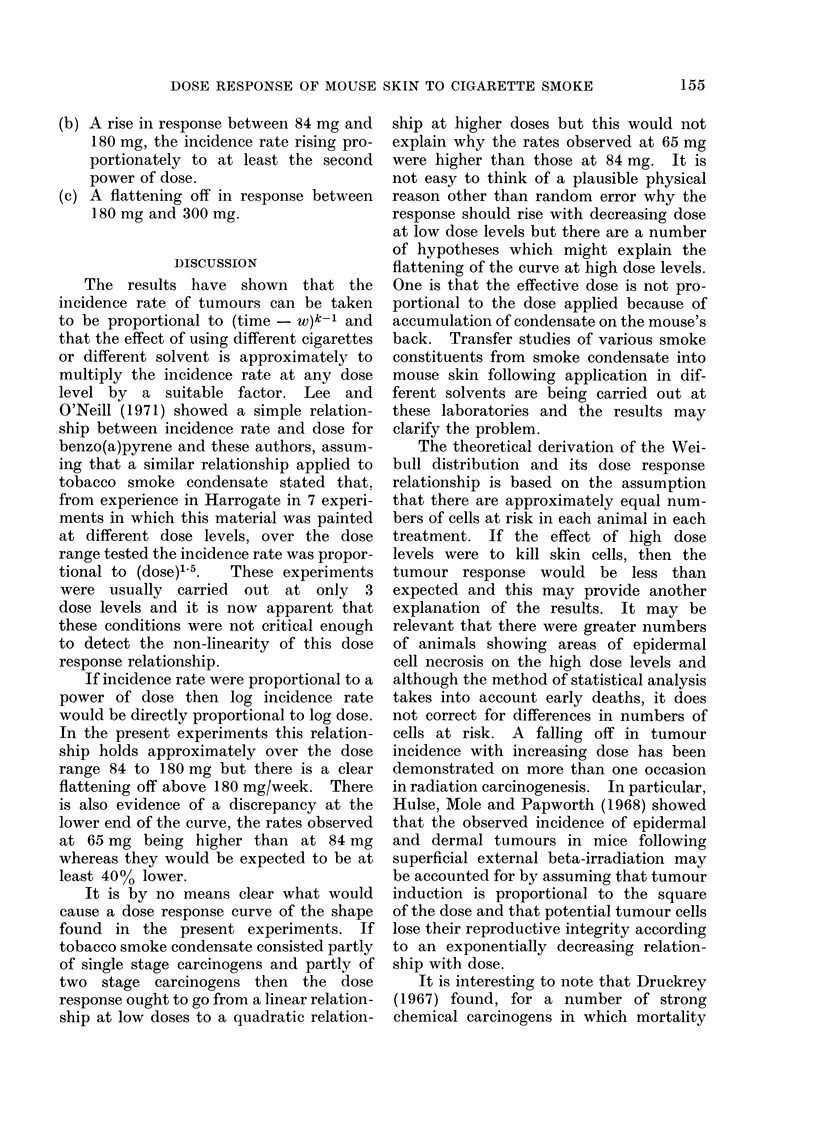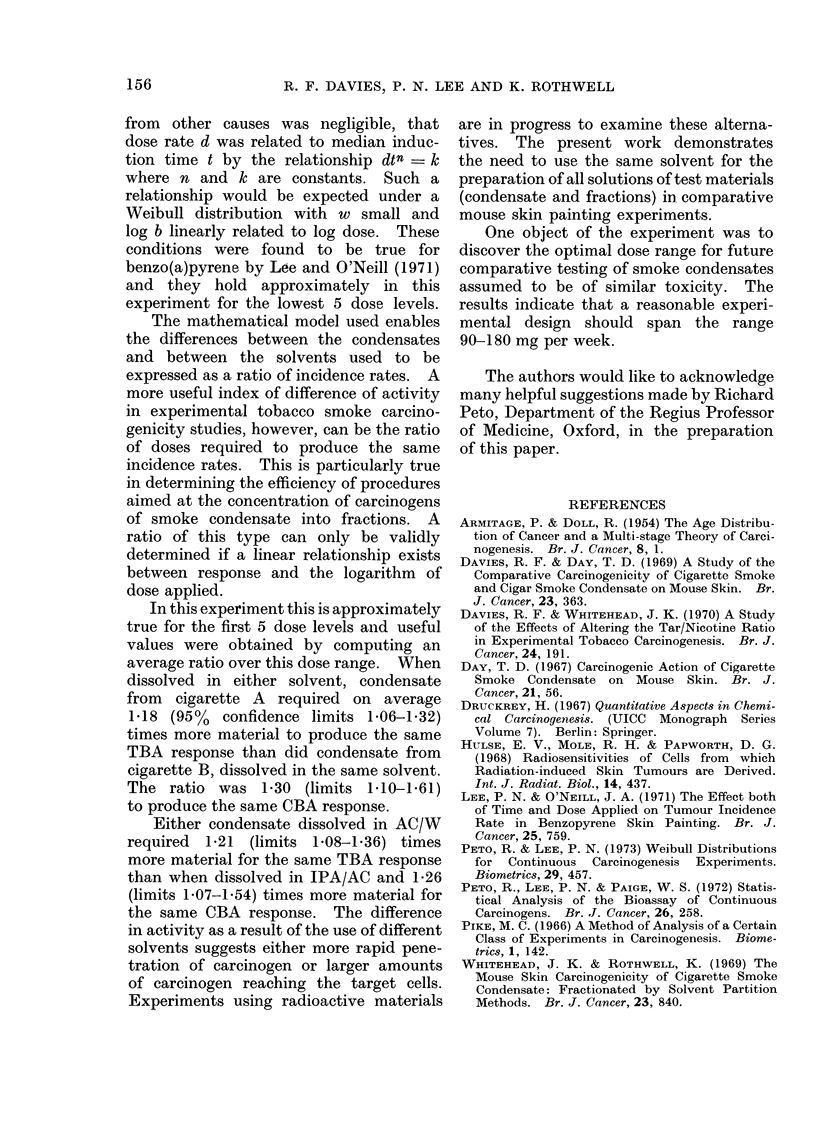# A Study of the Dose Response of Mouse Skin to Cigarette Smoke Condensate

**DOI:** 10.1038/bjc.1974.126

**Published:** 1974-08

**Authors:** R. F. Davies, P. N. Lee, K. Rothwell

## Abstract

Smoke condensate from two types of cigarette, dissolved in two solvents, has been applied regularly to the backs of mice at each of seven different dose levels. Treatment was continued 3 times weekly for up to 110 weeks, by which time 509 of the 1428 treated mice had developed skin tumours. The dependence of tumour incidence on age was adequately described by the Weibull distribution. The relation - ship between dose of smoke condensate and tumour incidence rate was, however, erratic. It was less regular than the simple relationship which has in previous work been found to obtain when the pure carcinogen benzo(a)pyrene is applied to mouse skin.


*Br. J. Cancer* (1974) **30**, 146

### A STUDY OF THE DOSE RESPONSE OF MOUSE SKIN TO CIGARETTE SMOKE CONDENSATE

R. F. DAVIES, P. N. LEE* AND K. ROTHWELL

*From the Tobacco Research Council Laboratories, Otley Road, Harrogate*

Received 26 September 1973. Accepted 3 May 1974

**Summary.—Smoke condensate from two types of cigarette, dissolved in two solvents, has been applied regularly to the backs of mice at each of seven different dose levels. Treatment was continued 3 times weekly for up to 110 weeks, by which time 509 of the 1428 treated mice had developed skin tumours. The dependence of tumour incidence on age was adequately described by the Weibull distribution. The relationship between dose of smoke condensate and tumour incidence rate was, however, erratic. It was less regular than the simple relationship which has in previous work been found to obtain when the pure carcinogen benzo(a)pyrene is applied to mouse skin.**

MOUSE skin tumour production as a result of the application of tobacco smoke condensate or its fractions is used extensively as an experimental model in the study of tobacco smoke carcinogenesis. In a large scale mouse skin painting experiment, Day (1967) showed that after allowance was made for the toxic effects of the painted material, the tumour yield was approximately proportional to the logarithm of the weekly dose of cigarette smoke condensate applied. This conclusion was, however, based on results from the application of only three dose levels of each material. The main purpose of the work described in this paper was to study the dose response relationship in greater detail, and to compare this relationship with that obtained previously for benzo(a)pyrene (Lee and O'Neill, 1971).

In many of the previous carcinogenicity experiments carried out at these laboratories (Day, 1967; Davies and Day, 1969; Whitehead and Rothwell, 1969; Davies and Whitehead, 1970), smoke condensate and fractions therefrom were dissolved in acetone/water 9 : 1 v/v (AC/W). Recently, with further development of the chemical fractionation of tobacco smoke condensate, many materials are insoluble in AC/W and have been dissolved in isopropyl alcohol/acetone 4 : 1 v/v (IPA/AC). A subsidiary purpose of the work described was to determine if there is any effect of the solvent on the mouse skin carcinogenicity of smoke condensate.

When tobacco smoke condensate is applied repetitively to the backs of mice not only does the carcinogenic insult increase as the amount of condensate is raised but there is also an increase in mortality due to local and systemic toxic effects to the animal until a percentage tumour yield is achieved which cannot be increased by applying larger amounts of condensate. If very large quantities are applied, moreover, the effective dose may be much less than the applied dose. The best dose levels to use in future assays of the carcinogenicity of cigarette smoke condensates are those at the upper end of the range in which the carcinogenic force is still strongly dependent on the applied dose, unless the toxic effects are

* Present address: Tobacco Research Council, Glen House, Stag Place, London SW1E 5AG.

so severe at this point that lower doses would actually yield higher percentages of tumour bearing animals. It was hoped that the present work would indicate the optimum practical dose range to apply in future assays.

### MATERIALS AND METHODS

*Cigarettes (A and B).*—Cigarettes (length 70 mm, circumference 25 mm, average weight 1·1 g) were specially manufactured from two different blends of flue-cured tobacco, packed in batches of 50 in vacuum-sealed tins and stored at 4°C, before use.

*Smoking procedures.*—The cigarettes were smoked in the automatic smoking machine described by Day (1967) using the same smoking parameters.

*Non-volatile whole smoke condensate (NVWSC).*—The cigarette smoke was condensed in the same type of traps and treated in the same way as previously described by Davies and Day (1969).

*Stored condensate.*—NVWSC collected over 4 weeks was combined, stored at −29°C for a further 4 weeks, dissolved with constant stirring in the appropriate solvent (AC/W or IPA/AC) and finally diluted to the appropriate volume with the same solvent prior to skin application.

*Mice.*—1908 female, albino mice were obtained from Carworth Europe, at 4–6 weeks of age, 4 weeks before first treatment.

*Details of treatment.*—The mice were randomly allocated to groups as follows:

1. 28 treatment groups of 51 mice in which a dose of stored condensate was applied in 0·3 ml of solvent 3 times a week. Each of the following 28 combinations (2 × 2 × 7) were tested.
   Cigarettes—A or B
   Solvents—AC/W or IPA/AC
   and Dose levels—65, 84, 108, 139, 180, 232 or 300 mg equivalent of NVWSC per week. (These dose levels are equally spaced on a log scale.)
2. 2 control groups of 180 mice in which 0·3 ml of solvent was applied 3 times a week—either AC/W or IPA/AC.
3. 1 control group of 120 mice completely untreated (apart from shaving).

Applications were made 3 times a week on Monday, Wednesday and Friday and continued for 110 weeks when the few surviving animals were killed.

*Recording of tumours and infiltrating carcinomata.*—Tumours of the treated area were recorded by visual inspection. The week of (first) tumour was taken as the week that a tumour was first observed on the living mouse, whether or not it later regressed or became malignant.

The criterion of malignancy adopted for tumours in the treated area was penetration of the muscle fibres of the panniculus carnosus and mice satisfying this criterion were said to have an infiltrating carcinoma. The week of infiltrating carcinoma was taken as the week of death of the animal.

*Other details.*—Procedures used for animal husbandry, skin shaving, the application of condensates, postmortem and histopathological examination of mouse skin were as previously described (Day, 1967; Davies and Day, 1969).

### RESULTS

The percentages of tumour bearing animals (TBA) and infiltrating carcinoma bearing animals (CBA) recorded at the end of the experiment are given in Tables IA and IB. Each CBA is, of course, also a TBA. A separate analysis of results for TBA and CBA was carried out because, although infiltrating carcinomata may be more relevant to human disease, more mice developed a tumour than developed an infiltrating carcinoma, so the tumour rates are estimated more accurately than the infiltrating carcinoma rates. Fig. 1 and 2 illustrate the percentage response to dose relationship for TBA and CBA respectively. These percentages obviously depend not only on the carcinogenic forces of the different treatments but also on the numbers of mice surviving into old age when the tumour incidence rates are highest. To correct for the effects of chance (or systematic) mortality on tumour yield, the dependence on treatment of the tumour incidence among the survivors at any age must be studied, rather than the dependence on treatment of the total tumour yield over all ages.

It has been suggested that the distribution of time to tumour can be satisfactorily approximated by either a lognormal

TABLE IA.—*Total Percentage of Tumour Bearing Animals by* 110 *weeks.* (51 *mice/treatment group*)

| | Dose level mg/week | | | | | | |
|---|---|---|---|---|---|---|---|
| | 65 | 84 | 108 | 139 | 180 | 232 | 300 |
| Cig. A(AC/W) | 14 | 10 | 12 | 28 | 45 | 31 | 51 |
| Cig. A(IPA/AC) | 20 | 16 | 28 | 35 | 47 | 51 | 49 |
| Cig. B(AC/W) | 16 | 10 | 16 | 31 | 51 | 61 | 65 |
| Cig. B(IPA/AC) | 20 | 22 | 33 | 43 | 59 | 63 | 69 |

| | | |
|---|---|---|
| Control AC/W | (180 mice) | 1 |
| Control IPA/AC | (180 mice) | 2 |
| Untreated Control | (120 mice) | 0 |

TABLE IB.—*Total Percentage of Infiltrating Carcinoma Bearing Animals by* 110 *weeks.* (51 *mice/treatment group*)

| | Dose level mg/week | | | | | | |
|---|---|---|---|---|---|---|---|
| | 65 | 84 | 108 | 139 | 180 | 232 | 300 |
| Cig. A(AC/W) | 0 | 2 | 0 | 4 | 6 | 18 | 28 |
| Cig. A(IPA/AC) | 2 | 0 | 8 | 4 | 14 | 14 | 22 |
| Cig. B(AC/W) | 6 | 0 | 2 | 6 | 14 | 22 | 18 |
| Cig. B(IPA/AC) | 2 | 2 | 10 | 12 | 28 | 28 | 28 |

| | | |
|---|---|---|
| Control AC/W | (180 mice) | 0 |
| Control IPA/AC | (180 mice) | 0 |
| Untreated Control | (120 mice) | 0 |

distribution (Day, 1967) or a Weibull distribution (Pike, 1966). Peto, Lee and Paige (1972) give reasons for preferring a Weibull distribution so this has been used for the subsequent analysis.

In the notation of Pike, no tumours will arise during the first $w$ weeks of treatment. After this minimum induction time, the incidence of tumours among the tumour-free survivors during week $t$ of the treatment is given by:

$$\text{incidence rate at week } t = bk(t - w)^{k-1}$$

which is a particular case of the Weibull distribution. $w$ and $k$ are independent of the carcinogenic insult which is measured by the parameter $b$. Armitage and Doll (1954) give an interpretation of the physical meaning of these parameters.

In order to find how $b$ is related to treatment, maximum likelihood estimates of a common $w$ and $k$ and a separate $b$ for each treatment subgroup were computed, using the method described by Peto and Lee (1973).* Tables IIa and IIb give the values of the parameters fitted for each subgroup.

In order to test the goodness of fit of these Weibull distributions to the data, the results for each subgroup were divided into 10 time periods. For each period the observed numbers of TBA and CBA were compared with the numbers expected using the fitted values of $b$, $w$ and $k$ given in Tables IIA and IIB. Each treatment group was analysed in this way and then the numbers were summed together within each time period to give the results displayed in Tables IIIA and IIIB. A $\chi$-squared statistic testing the overall goodness of fit for the 10 time periods is also given in these tables.

Inspection of the results from Tables IIIA and IIIB suggests that the Weibull distribution fits the data adequately.

* Peto and Lee (1973) used data from this experiment as an example of this method and suggested that $k$ should be a whole number. This recommendation has not been followed in the present paper as one aim of the work was to compare the form of the NVWSC dose/response relationship with the benzypyrene dose/response relationship reported by Lee and O'Neill (1971), who did not restrict $k$ to being a whole number.

FIG. 1.—Relationship between % tumour bearing animals and dose level.

Thus the carcinogenic effect in each subgroup can be described by the single parameter *b*. This parameter can be called the " relative incidence rate " as a value of *b* in one group *r* times higher than a value of *b* in another group implies that after any particular duration of treatment, the probability of the tumour-free animal getting a tumour in the near future is *r* times higher in the first group.

The next stage in the analysis is to consider the relationship of these relative

FIG. 2.—Relationship between % infiltrating carcinoma bearing animals and dose level.

incidence rates to cigarette type, to type of solvent and to dose applied.

To see whether there is a difference in the carcinogenicity of the two cigarette types, the 28 treatment groups were divided into 14 pairs of groups matched on dose level and solvent but differing in cigarette type.

If cigarette B is more carcinogenic, there should be a tendency within each pair of groups for the B group to suffer a higher relative incidence rate than the A group. Overall, the relative incidence rates did tend to be higher in the B groups, by an overall factor of 1·52 for TBA ($\chi^2 = 21{\cdot}8$ on 1 d.f., $P < 0{\cdot}001$) and by 1·63 for CBA ($\chi^2 = 8{\cdot}9$ on 1 d.f., $P < 0{\cdot}005$).

A similar argument, in which the 28 groups were divided into 14 pairs such that the two groups in one pair differed only with respect to solvent type, showed that a particular dose of a particular condensate tends to be more carcinogenic

TABLE IIA.—*Fitted Weibull Parameters. Tumour Bearing Animals* $w = 12 \cdot 26$ $k = 2 \cdot 81$ *values of* $b \times 10^6$

| | Dose level mg/week | | | | | | |
|---|---|---|---|---|---|---|---|
| | 65 | 84 | 108 | 139 | 180 | 232 | 300 |
| Cig. A(AC/W) | 1·04 | 0·68 | 0·87 | 1·97 | 5·90 | 5·15 | 11·95 |
| Cig. A(IPA/AC) | 1·62 | 0·98 | 2·46 | 3·66 | 8·46 | 9·26 | 11·29 |
| Cig. B(AC/W) | 1·06 | 0·91 | 1·39 | 3·46 | 11·22 | 13·55 | 16·34 |
| Cig. B(IPA/AC) | 1·66 | 1·70 | 3·31 | 4·57 | 10·90 | 13·30 | 17·26 |

TABLE IIB.—*Fitted Weibull Parameters. Infiltrating Carcinoma Bearing Animals.* $w = 36 \cdot 48$ $k = 3 \cdot 09$ *values of* $b \times 10^7$

| | Dose level mg/week | | | | | | |
|---|---|---|---|---|---|---|---|
| | 65 | 84 | 108 | 139 | 180 | 232 | 300 |
| Cig. A(AC/W) | 0·00 | 1·15 | 0·00 | 2·27 | 6·90 | 28·50 | 41·52 |
| Cig. A(IPA/AC) | 1·32 | 0·00 | 5·36 | 3·09 | 18·31 | 14·21 | 33·29 |
| Cig. B(AC/W) | 3·27 | 0·00 | 1·57 | 5·33 | 18·47 | 31·29 | 29·16 |
| Cig. B(IPA/AC) | 1·41 | 1·33 | 8·08 | 8·50 | 41·78 | 39·12 | 46·29 |

TABLE IIIA.—*Test of Goodness of Fit of the Parameters of Table IIa. Tumour Bearing Animals*

| Period in weeks | Observed | Expected |
|---|---|---|
| 0– 32 | 28 | 35·11 |
| 33– 40 | 56 | 48·45 |
| 41– 48 | 79 | 66·89 |
| 49– 56 | 81 | 78·30 |
| 57– 64 | 72 | 78·40 |
| 65– 72 | 67 | 73·50 |
| 73– 80 | 51 | 59·60 |
| 81– 88 | 35 | 37·90 |
| 89– 96 | 25 | 17·37 |
| 97–120 | 12 | 10·48 |

$\chi^2 = 12 \cdot 95$ on 9 d.f. Not significant.

TABLE IIIB.—*Test of Goodness of Fit of the Parameters of Table IIb. Infiltrating Carcinoma Bearing Animals*

| Period in weeks | Observed | | Expected | |
|---|---|---|---|---|
| 0– 32 | 0 | | 0·00 | |
| 33– 40 | 0 | 3 | 0·12 | 3·42 |
| 41– 48 | 3 | | 3·30 | |
| 49– 56 | 14 | | 11·81 | |
| 57– 64 | 25 | | 21·52 | |
| 65– 72 | 28 | | 29·50 | |
| 73– 80 | 19 | | 33·69 | |
| 81– 88 | 33 | | 24·78 | |
| 89– 96 | 17 | | 15·66 | |
| 97–120 | 11 | | 9·61 | |

$\chi^2 = 10 \cdot 54$ on 7 d.f. Not significant.

if dissolved in IPA/AC than if dissolved in AC/W. Using IPA/AC as solvent the incidence rates were overall 1·32 times higher for TBA ($\chi^2 = 9{\cdot}7$ on 1 d.f., $P < 0{\cdot}005$) and 1·34 times higher for CBA ($\chi^2 = 3{\cdot}6$ on 1 d.f., $P \simeq 0{\cdot}06$).

To study the shape of the dose response curve a similar argument was again used, but this time the division of the 28 groups of animals is into 4 lots of 7. In each lot the same substance was applied in the same solvent but at 7 different dose levels, and an average of the shapes of the 4 dose-response curves was obtained. (The statistical details of how this averaging was done, and of how the average effects of the cigarette and solvent differences were estimated, appear in Peto and Lee (1973).)

Fig. 3 and 4 present the dose-response relationships in graphical form for TBA and CBA respectively. The 4 separate dose response curves are given and so is their " average ". (The separate curves for CBA at the 3 lowest doses are omitted because they are based on so few infiltrating carcinomata that separately they are meaningless: the data for them may, however, be found in Table IIB.)

Tables IVA and IVB give for TBA and CBA respectively the " average " values of $b$ for the 7 dose levels with their approximate standard errors.

FIG. 3.—Relationship between the dose level and the relative incidence rate *b* of tumour bearing animals on a log/log scale.

FIG. 4.—Relationship between the dose level and the relative incidence rate *b* of infiltrating carcinoma bearing animals on a log/log scale.

TABLE IVA.—*Average Dose Response Relationship. Tumour Bearing Animals*

| Dose level mg/week | $b \times 10^6$ | S.E. |
|---|---|---|
| 65 | 1·39 | 0·23 |
| 84 | 1·13 | 0·21 |
| 108 | 2·06 | 0·31 |
| 139 | 3·56 | 0·42 |
| 180 | 9·51 | 0·94 |
| 232 | 10·65 | 1·04 |
| 300 | 14·87 | 1·36 |

TABLE IVB.—*Average Dose Response Relationship. Infitrating Carcinoma Bearing Animals*

| Dose level mg/week | $b \times 10^7$ | S.E. |
|---|---|---|
| 65 | 1·66 | 0·74 |
| 84 | 0·67 | 0·47 |
| 108 | 3·76 | 1·19 |
| 139 | 4·84 | 1·34 |
| 180 | 21·46 | 3·85 |
| 232 | 28·26 | 4·41 |
| 300 | 39·27 | 5·67 |

It is clear from these figures and Tables that, although there is a tendency for the response to increase with dose, the increase is not regular. (A test for the non-linearity of the relationship between log dose and log response shows that this apparent irregularity is not merely random fluctuation: for TBA, $\chi^2 = 25{\cdot}6$ on 5 d.f., ($P < 0{\cdot}001$) and for CBA $\chi^2 = 14{\cdot}9$ on 5 d.f. ($P < 0{\cdot}025$). The chief irregularity is the flattening off after the 180 mg dose level.)

As a test of the goodness of fit of the "no interaction" assumption that the 4 dose/response curves differ in shape only by random fluctuations, in Tables VA and VB the observed number of tumours in each treatment group are contrasted with the number expected if this hypothesis were true. Inspection of these Tables and also of Fig. 3 and 4 indicates that the 4 dose response curves

TABLE VA.—*Goodness of Fit of the "No Interaction" Model to the Data. Tumour Bearing Animals*

| | | Dose level mg/week | | | | | | |
|---|---|---|---|---|---|---|---|---|
| | | 65 | 84 | 108 | 139 | 180 | 232 | 300 |
| Cig. A(AC/W) | O | 7 | 5 | 6 | 14 | 23 | 16 | 26 |
| | E | 6·1 | 5·4 | 9·3 | 16·4 | 24·0 | 21·6 | 21·2 |
| Cig. A(IPA/AC) | O | 10 | 8 | 14 | 18 | 24 | 26 | 25 |
| | E | 7·5 | 8·0 | 10·1 | 15·1 | 23·2 | 25·8 | 28·5 |
| Cig. B(AC/W) | O | 8 | 5 | 8 | 16 | 26 | 31 | 33 |
| | E | 10·4 | 6·1 | 11·7 | 16·2 | 21·7 | 24·1 | 29·8 |
| Cig. B(IPA/AC) | O | 10 | 11 | 17 | 22 | 30 | 32 | 35 |
| | E | 11·0 | 9·5 | 13·9 | 22·3 | 34·1 | 33·5 | 39·6 |

$\chi^2 = 14{\cdot}83$ on 19 d.f. Not significant.

TABLE VB.—*Goodness of Fit of the "No Interaction" Model to the Data. Infiltrating Carcinoma Bearing Animals*

| | | Dose level mg/week | | | | | | |
|---|---|---|---|---|---|---|---|---|
| | | 65 | 84 | 108 | 139 | 180 | 232 | 300 |
| Cig. A(AC/W) | O | 0 | 1 | 0 | 2 | 3 | 9 | 14 |
| | E | 0·8 | 0·4 | 1·9 | 2·7 | 5·7 | 5·4 | 8·1 |
| Cig. A(IPA/AC) | O | 1 | 0 | 4 | 2 | 7 | 7 | 11 |
| | E | 1·1 | 0·6 | 2·4 | 2·7 | 6·9 | 11·6 | 10·9 |
| Cig. B(AC/W) | O | 3 | 0 | 1 | 3 | 7 | 11 | 9 |
| | E | 1·6 | 0·4 | 2·5 | 2·8 | 8·3 | 10·1 | 12·4 |
| Cig. B(IPA/AC) | O | 1 | 1 | 5 | 6 | 14 | 14 | 14 |
| | E | 1·6 | 0·7 | 3·3 | 4·8 | 10·1 | 14·0 | 16·6 |

$\chi^2 = 25{\cdot}29$ on 19 d.f. Not significant.

may not be completely parallel and may tend to converge at the top dose levels. Thus the " no interaction " model, which postulates that the effect of type of cigarette, type of solvent used and dose of condensate applied are all independent factors which act together multiplicatively, though a reasonable approximate way of stating the results, may to some extent be an over simplification. As the effects of cigarette type and solvent type are obviously much smaller than the effects of dose, and also as the dose response is rather unusual above 180 mg, the data are not really adequate to test this multiplicative hypothesis critically.

Lee and O'Neill (1971) showed for benzo(a)pyrene that $b$ was proportional to dose$^2$ over the dose range 6 $\mu g$ to 48 $\mu g$ per week. In the experiment described in this paper it had been hoped to see whether a similar relationship held for NVWSC. However for reasons to be considered in the Discussion it was clear that the 232 mg and 300 mg dose levels could not be included in any simple dose response relationship.

We therefore tested whether $b$ could be taken as proportional to dose$^a$ by fitting this relationship to the first 5 dose levels only. The maximum likelihood values of $a$ found were 2·23 for TBA and 3·28 for CBA. Values of $a$ of 2 and 2·5 for TBA were not much worse fits to the data than the fitted value ($\chi^2 = 2{\cdot}87$ and 4·11 respectively, each on 1 d.f.), but values of 1, 1·5 and 3 were clearly unacceptable ($\chi^2 = 88{\cdot}5$, 30·3 and 31·6). Similarly for CBA values of $a$ of 3 and 3·5 were reasonable fits ($\chi^2 = 0{\cdot}70$ and 0·44) but 2 was not ($\chi^2 = 16{\cdot}4$) and 2·5 and 4 were doubtful ($\chi^2 = 5{\cdot}8$ and 4·4).

Although this shows that over the dose range 65 mg to 180 mg the relationship between relative incidence rate and dose for both TBA and CBA is far better approximated by a square or cube law than by a simple linear law, it was not possible to show that the best fitting laws of this form fitted the data completely adequately. Tables VIA and VIB give, respectively, the numbers of TBA and CBA expected under this best fitting law, for comparison with those observed. Although the fit was made to the lowest 5 dose levels it has been extrapolated to 232 mg and 300 mg to demonstrate the falling off in response at these levels.

From these tables it can be seen that the response at 65 mg was higher than expected and the rise between 84 mg and 180 mg somewhat steeper than expected.

The dose response can thus be summarized by three characteristics:

(a) A slight drop in response between 65 and 84 mg.

TABLE VIA.—*Goodness of Fit to Dose Response Law of the Form* b *Proportional to Dose*$^a$ *Fitted to the Lowest* 5 *Dose Levels. Tumour Bearing Animals* ( )* = *extrapolated value.* $a = 2{\cdot}225$

| | Dose level mg/week | | | | | | |
|---|---|---|---|---|---|---|---|
| | 65 | 84 | 108 | 139 | 180 | 232 | 300 |
| Observed | 35 | 29 | 45 | 70 | 103 | 105 | 119 |
| Expected | 20·2 | 37·1 | 54·3 | 86·0 | 84·4 | (135·1)* | (197·6)* |

TABLE VIB.—*Goodness of Fit to Dose Response Law of the Form* b *Proportional to Dose*$^a$ *Fitted to the Lowest* 5 *Dose Levels. Infitrating Carcinoma Bearing Animals* ( )* = *extrapolated value.* $a = 3{\cdot}276$

| | Dose level mg/week | | | | | | |
|---|---|---|---|---|---|---|---|
| | 65 | 84 | 108 | 139 | 180 | 232 | 300 |
| Observed | 5 | 2 | 10 | 13 | 31 | 41 | 48 |
| Expected | 1·9 | 4·4 | 8·8 | 20·2 | 25·6 | (61·7)* | (117·2)* |

(b) A rise in response between 84 mg and 180 mg, the incidence rate rising proportionately to at least the second power of dose.
(c) A flattening off in response between 180 mg and 300 mg.

### DISCUSSION

The results have shown that the incidence rate of tumours can be taken to be proportional to (time — $w)^{k-1}$ and that the effect of using different cigarettes or different solvent is approximately to multiply the incidence rate at any dose level by a suitable factor. Lee and O'Neill (1971) showed a simple relationship between incidence rate and dose for benzo(a)pyrene and these authors, assuming that a similar relationship applied to tobacco smoke condensate stated that, from experience in Harrogate in 7 experiments in which this material was painted at different dose levels, over the dose range tested the incidence rate was proportional to $(\text{dose})^{1\cdot5}$. These experiments were usually carried out at only 3 dose levels and it is now apparent that these conditions were not critical enough to detect the non-linearity of this dose response relationship.

If incidence rate were proportional to a power of dose then log incidence rate would be directly proportional to log dose. In the present experiments this relationship holds approximately over the dose range 84 to 180 mg but there is a clear flattening off above 180 mg/week. There is also evidence of a discrepancy at the lower end of the curve, the rates observed at 65 mg being higher than at 84 mg whereas they would be expected to be at least 40% lower.

It is by no means clear what would cause a dose response curve of the shape found in the present experiments. If tobacco smoke condensate consisted partly of single stage carcinogens and partly of two stage carcinogens then the dose response ought to go from a linear relationship at low doses to a quadratic relationship at higher doses but this would not explain why the rates observed at 65 mg were higher than those at 84 mg. It is not easy to think of a plausible physical reason other than random error why the response should rise with decreasing dose at low dose levels but there are a number of hypotheses which might explain the flattening of the curve at high dose levels. One is that the effective dose is not proportional to the dose applied because of accumulation of condensate on the mouse's back. Transfer studies of various smoke constituents from smoke condensate into mouse skin following application in different solvents are being carried out at these laboratories and the results may clarify the problem.

The theoretical derivation of the Weibull distribution and its dose response relationship is based on the assumption that there are approximately equal numbers of cells at risk in each animal in each treatment. If the effect of high dose levels were to kill skin cells, then the tumour response would be less than expected and this may provide another explanation of the results. It may be relevant that there were greater numbers of animals showing areas of epidermal cell necrosis on the high dose levels and although the method of statistical analysis takes into account early deaths, it does not correct for differences in numbers of cells at risk. A falling off in tumour incidence with increasing dose has been demonstrated on more than one occasion in radiation carcinogenesis. In particular, Hulse, Mole and Papworth (1968) showed that the observed incidence of epidermal and dermal tumours in mice following superficial external beta-irradiation may be accounted for by assuming that tumour induction is proportional to the square of the dose and that potential tumour cells lose their reproductive integrity according to an exponentially decreasing relationship with dose.

It is interesting to note that Druckrey (1967) found, for a number of strong chemical carcinogens in which mortality from other causes was negligible, that dose rate $d$ was related to median induction time $t$ by the relationship $dt^n = k$ where $n$ and $k$ are constants. Such a relationship would be expected under a Weibull distribution with $w$ small and log $b$ linearly related to log dose. These conditions were found to be true for benzo(a)pyrene by Lee and O'Neill (1971) and they hold approximately in this experiment for the lowest 5 dose levels.

The mathematical model used enables the differences between the condensates and between the solvents used to be expressed as a ratio of incidence rates. A more useful index of difference of activity in experimental tobacco smoke carcinogenicity studies, however, can be the ratio of doses required to produce the same incidence rates. This is particularly true in determining the efficiency of procedures aimed at the concentration of carcinogens of smoke condensate into fractions. A ratio of this type can only be validly determined if a linear relationship exists between response and the logarithm of dose applied.

In this experiment this is approximately true for the first 5 dose levels and useful values were obtained by computing an average ratio over this dose range. When dissolved in either solvent, condensate from cigarette A required on average 1·18 (95% confidence limits 1·06–1·32) times more material to produce the same TBA response than did condensate from cigarette B, dissolved in the same solvent. The ratio was 1·30 (limits 1·10–1·61) to produce the same CBA response.

Either condensate dissolved in AC/W required 1·21 (limits 1·08–1·36) times more material for the same TBA response than when dissolved in IPA/AC and 1·26 (limits 1·07–1·54) times more material for the same CBA response. The difference in activity as a result of the use of different solvents suggests either more rapid penetration of carcinogen or larger amounts of carcinogen reaching the target cells. Experiments using radioactive materials are in progress to examine these alternatives. The present work demonstrates the need to use the same solvent for the preparation of all solutions of test materials (condensate and fractions) in comparative mouse skin painting experiments.

One object of the experiment was to discover the optimal dose range for future comparative testing of smoke condensates assumed to be of similar toxicity. The results indicate that a reasonable experimental design should span the range 90–180 mg per week.

The authors would like to acknowledge many helpful suggestions made by Richard Peto, Department of the Regius Professor of Medicine, Oxford, in the preparation of this paper.

### REFERENCES

ARMITAGE, P. & DOLL, R. (1954) The Age Distribution of Cancer and a Multi-stage Theory of Carcinogenesis. *Br. J. Cancer*, **8**, 1.

DAVIES, R. F. & DAY, T. D. (1969) A Study of the Comparative Carcinogenicity of Cigarette Smoke and Cigar Smoke Condensate on Mouse Skin. *Br. J. Cancer*, **23**, 363.

DAVIES, R. F. & WHITEHEAD, J. K. (1970) A Study of the Effects of Altering the Tar/Nicotine Ratio in Experimental Tobacco Carcinogenesis. *Br. J. Cancer*, **24**, 191.

DAY, T. D. (1967) Carcinogenic Action of Cigarette Smoke Condensate on Mouse Skin. *Br. J. Cancer*, **21**, 56.

DRUCKREY, H. (1967) *Quantitative Aspects in Chemical Carcinogenesis.* (UICC Monograph Series Volume 7). Berlin: Springer.

HULSE, E. V., MOLE, R. H. & PAPWORTH, D. G. (1968) Radiosensitivities of Cells from which Radiation-induced Skin Tumours are Derived. *Int. J. Radiat. Biol.*, **14**, 437.

LEE, P. N. & O'NEILL, J. A. (1971) The Effect both of Time and Dose Applied on Tumour Incidence Rate in Benzopyrene Skin Painting. *Br. J. Cancer*, **25**, 759.

PETO, R. & LEE, P. N. (1973) Weibull Distributions for Continuous Carcinogenesis Experiments. *Biometrics*, **29**, 457.

PETO, R., LEE, P. N. & PAIGE, W. S. (1972) Statistical Analysis of the Bioassay of Continuous Carcinogens. *Br. J. Cancer*, **26**, 258.

PIKE, M. C. (1966) A Method of Analysis of a Certain Class of Experiments in Carcinogenesis. *Biometrics*, **1**, 142.

WHITEHEAD, J. K. & ROTHWELL, K. (1969) The Mouse Skin Carcinogenicity of Cigarette Smoke Condensate: Fractionated by Solvent Partition Methods. *Br. J. Cancer*, **23**, 840.